# Supplementary material for: Chromothripsis during telomere crisis is independent of NHEJ, and consistent with a replicative origin
Source: Genome Res. 2019 May;29(5):737–49. doi: 10.1101/gr.240705.118 (PMC6499312; doi:10.1101/gr.240705.118)
Supplement: Supplemental Material [file supp_gr.240705.118_Supplemental_file_1.zip › contigs/annotated_contigs/DB41/contig.2.DB41_length_724_mean_cov_3.52209944751.docx]

**DB41_length_724_mean_cov_3.52209944751**

```
ATGTTAGCAAGCGGCTTCTTGATTGAAGTGAGGAT|CACTAAAACATGATAATGGTTCTACTTACAATAGCTGTTTTAACAGGAAATCT
                                    >chr5:130799632-130800055 + E=1e-227
TCAAAAGCACATCTACAAATAAGCACATCCACTAATAAGCTTATTCACCTTCCATTGAGTTGAATTCTATCAGCTAATTTGGAGAACTG

ATCTGGAAGTCTTCTCTGTTTTATGACACCCTCAGGAGTAACAGAAACTTCACAGAGAGAATATGTGTCGGATGCACCGGTCAAACCAA

ATTCATGAACAGCATGAAAAACTACTTCTTTAGCTGTGGTGACTATACTGATGATAAGGTAGCAACTTTGCTGATCCACTTTGAAAACT

CTTATAACTTGCTCAGGGATATCTACATAAATAGAAAGATATGCTTTGTTATTTTTTAAAAAAATCACTTTTAAACCCTTCAAAAAGCA

ACGTGAAACA|CAGT|AAGGCCAAAAGTGGGAGGCAAACAGAGTGAGTTAAGGTTCAGATGTAGTCCAGGTTCAGTCTGTGCAGGCCAT
           >chr5:129179993-129180263 + E=1e-150
TACTGGCCACTCTGAGGGTTTTGGTCTTGTGGCAGGAGGCAATATCCATGTCTTCATTGTGACAAGAGTGGTGGCAGAGATGTATGAAT

GAAAGAGACATACTGAAAATCAACAGGATTTTCTAAGGTAGCCAAATCAATAGGATTTCCTAACATTAGAGGTGTGTCAGTGTTAAGAG

TGAAAGAATAATAGG
```
